# Screening of microorganisms from Antarctic surface water and cytotoxicity metabolites from Antarctic microorganisms

**DOI:** 10.1002/fsn3.273

**Published:** 2015-09-29

**Authors:** Lanhong Zheng, Kangli Yang, Jia Liu, Mi Sun, Jiancheng Zhu, Mei Lv, Daole Kang, Wei Wang, Mengxin Xing, Zhao Li

**Affiliations:** ^1^ Key Laboratory for Sustainable Utilization of Marine Fisheries ResourcesMinistry of AgricultureYellow Sea Fisheries Research InstituteChinese Academy of Fishery SciencesQingdao266071China; ^2^ Medical CollegeQingdao UniversityQingdao266021China

**Keywords:** Antarctic surface water, cytotoxicity, fermented active products, microorganism screening, MTT method

## Abstract

The Antarctic is a potentially important library of microbial resources and new bioactive substances. In this study, microorganisms were isolated from surface water samples collected from different sites of the Antarctic. 3‐(4,5‐dimethyl‐2‐thiazolyl)‐2,5‐diphenyl‐2H‐tetrazolium bromide (MTT) assay‐based cytotoxicity‐tracking method was used to identify Antarctic marine microorganism resources for antitumor lead compounds. The results showed that a total of 129 Antarctic microorganism strains were isolated. Twelve strains showed potent cytotoxic activities, among which a Gram‐negative, rod‐shaped bacterium, designated as N11‐8 was further studied. Phylogenetic analysis based on 16S rRNA gene sequence showed that N11‐8 belongs to the genus *Bacillus*. Fermented active products of N11‐8 with molecular weights of 1–30 kDa had higher inhibitory effects on different cancaer cells, such as BEL‐7402 human hepatocellular carcinoma cells, U251 human glioma cells, RKO human colon carcinoma cells, A549 human lung carcinoma cells, and MCF‐7 human breast carcinoma cells. However, they displayed lower cytotoxicity against HFL1 human normal fibroblast lung cells. However, they displayed lower cytotoxicity against HFL1 human normal fibroblast lung cells. Microscopic observations showed that the fermented active products have inhibitory activity on BEL‐7402 cells similar to that of mitomycin C. Further studies indicated that the fermented active products have high pH and high thermal stability. In conclusion, most strains isolated in this study may be developed as promising sources for the discovery of antitumor bioactive substances. The fermented active products of Antarctic marine *Bacillus* sp. N11‐ 8 are expected to be applied in the prevention and treatment of cancer.

## Introduction

Because of the unique geographic location and harsh natural environment — including low temperature, drought, and strong radiation — the polar region has not been polluted by humans and retains the original state. The Arctic and Antarctic have become not only an important place for recording the historical evolution process of earth system, but also a place of new microbial species resource (Anderson 1995; Gosink et al. 1998). The special habitat leads to unique molecular mechanisms and physiological and biochemical characteristics in genetic composition, properties, and metabolic regulation of polar microorganism. Microbiologists have conducted a lot of research work in the polar region, which has proved that the polar microorganisms in these special environments has a broad prospect in the development of novel drugs, new enzyme models, health food and other basic research and applications (Humphry et al. 2001; Shivaji et al. 2005). Polar region, therefore, becomes a potential, important microbial resources database, and a new bioactive substances and microbial potential provenance (Alan et al. 2000; Van Trappen et al. 2004; Wu et al. 2013).

In recent years, a number of polar microorganisms and the related metabolites were found to possess antitumor activity. Because of the increasing concern on marine and extreme environmental microbial resources, more and more compounds with antitumor activity are being identified from polar microorganisms (Zeng et al. 2008). Antibacterial activity of the Antarctic bacterium *Janthinobacterium* sp. SMN 33.6 was studied against multiresistant Gram‐negative bacteria (Asencioa et al. 2013). 259 strains were isolated from the samples collected from the Antarctic soil and the South Ocean water, 11% of which showed strong antitumor activity. The results showed that the Antarctic microorganisms have a good potential for bioactive metabolite research (Zhu et al. 2006; Ding et al. 2014). Even so, the number of marine antineoplastic drugs is still not comparative to antitumor drugs of terrestrial sources. In this study, a large number of microorganisms were isolated from surface water samples obtained from different sites in the Antarctic. A MTT assay‐based antitumor activity‐tracking method was used to identify new Antarctic marine microorganism resources for antitumor lead compounds.

## Materials and Methods

### Sample collection and isolation of Antarctic marine microorganisms

No specific permissions were required for the collection of sample from the location. The field studies did not involve endangered or protected species. The Antarctic surface seawater samples were collected from several different stations in Antarctic in March 2012 (Table 1). The surface water was filtered using 0.22 *μ*m membranes, and then the membranes were preserved in sterile vials containing 20% glycerol with Nansen bottles at −80°C before use.

**Table 1:** Antarctic surface seawater samples collected from several different stations in Antarctic

Station	Latitude	Longitude	Quantity of sea surface water was filtered (mL)
5#	**6002**	6002	200
6#	**6002**	5902	200
11#	6001	5402	200
16#	6100	6458	200
18#	6100	6257	200
26#	**6100**	5456	200
35#	6201	6301	400
39#	**6303**	6659	400
49#	6234	6158	200
51#	**6100**	6657	400

Provide the source of bacteria.

The microorganism strains were isolated by plating 200 *μ*L of different dilutions of the samples preserved in the sterile vials containing 20% glycerol (from 10^−1^ to 10^−7^) on 2216E seawater‐based medium and beef extract‐peptone medium agar plates in triplicates, respectively. The inoculated plates were incubated at 4, 15, and 25°C, respectively, for 3–6 days. Colonies arising on all solid plates were selected based on their physiological features and morphological characteristic — including rate of growth, shape, size pigmentation, and margin (Button et al. 1993).

### Inoculum, fermentation and screening of bioactive product preparation

The basal medium used for the production of antitumor products consisted of 1.0% tryptone, 0.3% beef extract, and 0.5% NaCl (pH 6.5–7.0). Incubation was carried out in a rotary shaker at 25°C and 180–200 rpm. A loopful of cells from a slant was transferred to 3 mL of the above mentioned sterile medium in a 12 mL test tube and incubated at 25°C and 200 rpm for 24 h. This was used as the inoculum. Fermentation was carried out in 250‐mL Erlenmeyer flasks, each containing 50 mL of sterile production medium. The medium was inoculated with 4–10% (v/v) of the old culture. The inoculated flasks were kept on a rotary shaker (Thermo, Waltham, Massachusetts, America) at 25°C and 200 rpm for 48 h (Button et al. 1993). The fermented broth was collected and centrifuged at 11,180 g for 15 min, using a centrifuge (Hitachi, Eastportcity, Honshu, Japan). After centrifugation, the supernatant and the microorganism precipitation were separately collected. Microorganism precipitation was resuspended in 20 mL of 20 mmol/L PBS solution, and broken by ultrasonication for 10 min. The supernatants were collected after centrifugation. Extracellular and intracellular fermentation products of each strain were filtered using 0.22 *μ*m membrane, packed in tubules, and frozen at −20°C, prepared as the sample to be tested for its antitumor activity.

### Cell lines and culture conditions

BEL‐7402 human hepatocellular carcinoma cells, RKO human colon carcinoma cells, A549 human lung carcinoma cells, U251 human glioma cells, MCF‐7 human breast carcinoma cells, and HFL1 human normal fibroblast lung cells were provided by the Cell Bank of Chinese Academy of Sciences (Shanghai, China). BEL‐7402, RKO, A549, U251, and MCF‐7 cells were cultured in DMEM (Hyclone, South Logan, Utah, America) supplemented with 10% heat‐inactivated fetal bovine serum (Gibco, California, America), 100 U/mL of penicillin, and 100 mg/mL streptomycin (Sigma, St. Louis, Missouri, America). HFL1 cells were cultured in F12K (Hyclone) supplemented with 10% heat‐inactivated fetal bovine serum, 100 U/mL of penicillin, and 100 mg/mL streptomycin. Cells were grown in a carbon dioxide incubator (Thermo Forma, Waltham, Massachusetts, America) under a humidified atmosphere containing 5% CO_2_ at 37°C (Fogh et al. 1977).

### The screening of antitumor fermentation products from isolated strains

The inhibitory effects of fermentation products from the isolated strains on the viability of BEL‐7402, RKO, A549, U251, MCF‐7 cells, and normal HFL1 cells were evaluated using the MTT assay (Mosmann 1983). Some improvement was made in our study. In brief, the above cancer cells (2.2 × 10^4^ CFU/mL) in 180 *μ*L of DMEM culture media were seeded into each well on 96‐well microplates and cultured for 24 h. Then, 20 *μ*L SBP with certain concentrations of the fermentation products were added to the media. After incubation for 48 h, MTT solution (20 *μ*L, 0.5 mg/mL) was added to each well and the cells were cultured for another 4 h at 37°C. The media were removed and 150 *μ*L DMSO was added to dissolve the formazan crystals. The OD_570_ was measured with an Infinite M200 PRO microplate reader (TECAN Group Ltd, Mannerdorf, Switzerland) with subtraction of the background absorbance. All experiments were performed in triplicate. The cytotoxicity of the fermentation products from the isolated strains was expressed as inhibition ratio of cell viability (IR). IR = [(A_570_ value of the control‐A_570_ value of the experimental samples)/A_570_ value of the control] × 100%.

### Morphological, physiological and biochemical characteristics of strain N11‐8

Cell morphology was determined by SEM (scanning electron microscopy). The cells were grown onto poly‐l‐lysine‐coated coverslips in plate for 24 h to allow firm attachment. The cells were fixed with glutaraldehyde. After overnight fixation at 4°C, the coverslips were dehydrated using ethanol and dried in a critical point dryer. Cells on coverslips were coated with gold and analyzed, using S‐3400N SEM (Hitachi).

Standard protocols (Tindall et al. 2007) were used to assess oxidase activities. Activities of constitutive enzymes, substrate oxidation, carbon source utilization, and other physiological properties were determined using the API 20E and API 20NE, the API ZYM strips (bioMe′rieux, Lyon, France), and the Gram‐negative MicroPlates (Biolog, Hayward, California, America), according to the manufacturer's instructions.

### Phylogenetic analysis

Chromosomal DNA of the strain N11‐8 was extracted (Ausubel et al. 1995) and the 16S rRNA gene was amplified by PCR, (Biometra, Göttingen, Germany), using the universal primers 27f 5′‐AGAGTTTGATCCTGGTCAG‐3′ and 1492r 5′‐CGGCTACCTTGTTACGAC‐3′ (Weisburg et al. 1991). Purified PCR products were ligated into pMD 18‐T (TaKaRa, Minami kusatsu, Japan), according to the manufacturer's instructions. Sequencing reactions were carried out using ABI BigDye 3.1 Sequencing kits (Applied BioSystems, San Francisco, California, America) and an automated DNA sequencer (model ABI3730; Applied BioSystems). The near‐complete 16S rRNA gene sequence of the strain N11‐8 was submitted to GenBank/EMBL to search for the similar sequences using the BLAST algorithm. The identification of phylogenetic neighbors and the calculation of pairwise 16S rRNA gene sequence similarities were achieved using the EzTaxon server (http://www.eztaxon.org/) (Chun et al. 2007). The sequences were aligned using CLUSTAL X1.8 (Thompson et al. 1997). Phylogenetic trees were constructed using the neighbour‐joining (Fig. 2) methods implemented in the program MEGA version 6 (Tamura et al. 2013). In each case, bootstrap values were calculated based on 1000 replicates.

### Preparation of the active substance

For the production of secondary metabolites, the Antarctic marine Bacillus sp. N11‐8 was cultured as above. Fermentation products were centrifuged at 10,000 g for 15 min at 4°C, and the supernatant was then collected. The supernatant was ultrafiltered first using a 50 kDa molecular weight cutoff membrane, then with a 30 kDa molecular weight cutoff membrane, and finally with a 1 kDa molecular weight cutoff membrane. The filtrate with different molecular weight was determined for their antitumor activity. The fraction with highest cytotoxicity was collected for further experiments.

### Analysis of inhibitory effect of the fermentation products of Antarctic microorganism N11‐8 on the proliferation of various tumor cells

The inhibitory effects of the fermentation products of the isolated strains on the viability of BEL‐7402, RKO, A549, U251, MCF‐7 cells, and normal HFL1 cells were evaluated by MTT assay as described above. The IC_50_ value was defined as the concentration causing a 50% reduction in cell viability.

### Morphological changes in BEL‐7402 cells treated with the fermentation products of Antarctic microorganism N11‐8

The BEL‐7402 cells (2.2 × 10^4^ CFU/mL) in 180 *μ*L of DMEM culture media were seeded into each well on 96‐well microplates and cultured in a constant temperature incubator at 37°C and 5% CO_2_ for 24 h. After incubation with the fermentation products for 48 h, the morphology and number of tumor cells were observed and counted, respectively, under an inverted phase contrast microscope (Nikon, Tokyo, Japan).

### Acid stability analysis of the fermentation products of Antarctic microorganism N11‐8

The pH of 150 *μ*g/mL fermentation products of Antarctic microorganism N11‐8 (molecular weight 1–30 kDa) was adjusted to 2, 4, 6, 8, 10, or 12, placed at room temperature for 24 h, and then adjusted backed to the original pH value. The cytotoxicity of the fermentation products on BEL‐7402 tumor cells was determined by MTT method. Each experiment was repeated for three times.

### Thermal stability analysis of the fermentation products of Antarctic microorganism N11‐8

One hundred and fifty *μ*g/mL fermentation product of Antarctic microorganism N11‐8 (molecular weight: 1–30 kDa) were placed at 40, 60, 80, 100, or 121°C for 30 min. After rapid cooling, the cytotoxicity of the fermentation products on BEL‐7402 tumor cells was determined by MTT method. Each experiment was repeated for three times.

### Statistical analysis

All of the tests were conducted in triplicate, and the experimental data were expressed as the mean ± SD. The statistical significance of the values between the control and the treated groups was determined by a paired *t*‐test; *P* < 0.05 was considered to be statistically significant.

## Results and Discussion

### Isolation of Antarctic microorganisms

Isolation and cultivation of a new marine microorganism may be a shortcut to discover novel natural products. A variety of pretreatment methods, for example physical and chemical enrichment, are employed to facilitate the isolation of specific marine microorganisms, especially less abundant bacteria [13, 14]. Using 2216E seawater‐based medium and beef extract‐peptone culture medium, a total of 129 microorganism strains were isolated from the samples collected from 10 different stations in Antarctic (Table 2). In the process of acquisition of Antarctic surface seawater samples, the microorganisms were retained in the membrane using a seawater filter. The enrichment process of microorganisms facilitated the storage, carrying, and is convenient for microorganism screening and follow‐up study.

**Table 2:** Separation of microorganisms from the surface seawater samples from different stations in Antarctic

Station	Latitude	Longitude	Microbial quantity (Strain)
5#	**6002**	6002	11
6#	**6002**	5902	13
11#	6001	5402	15
16#	6100	6458	13
18#	6100	6257	12
26#	**6100**	5456	12
35#	6201	6301	10
39#	**6303**	6659	14
49#	6234	6158	12
51#	**6100**	6657	17

Provide the source of bacteria.

### The screening of metabolites of Antarctic microorganisms with antitumor activity by MTT

The fermentation products of 129 strains of Antarctic microorganisms were screened for antitumor activity using MTT method. The inhibition rates of two Antarctic microorganism strains were more than 50% on BEL‐7402 human hepatocellular carcinoma cells; the inhibition rates of five strains were more than 30% (Table 3); The fermentation products of strain N11‐8 (No. 8) that was isolated from station 11# in the Antarctic showed 68.1% of IR on BEL‐7402 cells. According to the results, there are microbial germplasm resources for producing antitumor bioactive substances from the surface waters in Antarctic.

**Table 3:** Antitumor activity (means ± SD) of the fermented products of Antarctic microorganisms

Microbial strain number	Inhibition rate on BEL‐7402 cell (%)
N5‐6	31.6 ± 5.12^a^
N6‐2	43.2 ± 5.96^b^
N11‐8	68.1 ± 7.42^c^
N16	65.5 ± 7.12^c^
N18‐2	32.3 ± 4.85^a^
N26‐7	34.8 ± 4.13^a^
N49‐1	35.7 ± 5.02^a^

Data within the same column with different superscripts are significantly different (*P* < 0.05).

### Phenotypic and phylogenetic characterization of Antarctic microorganism N11‐8

**Figure 1:**
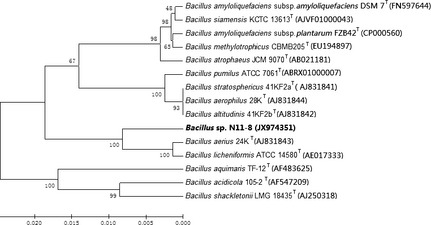
Phylogenetic dendrogram of *Bacillus* sp. N11‐8 and its related species based on 16S rRNA gene sequence similarities. The tree was constructed using the neighbour‐joining method implemented in the program MEGA version 6. Bar, 0.01 nt substitutions per site.

We isolated a new strain, N11‐8, from the surface water of Antarctic. The near‐complete 16S rRNA gene sequence (1434 nt) of strain N11‐8 was uploaded to GeneBank (GenBank accession number JX974351). Phylogenetic analysis based on the 16S rRNA sequence indicated that strain N11‐8 belonged to the genus *Bacillus*, with the highest sequence similarities to *Bacillus licheniformis* ATCC14580T (98.67%), *Bacillus aerius* 24kT (98.6%), *Bacillus siamensis* KCTC13613T (97.7%), *Bacillus amyloliquefaciens* subsp. *amylol* DSM7T (97.63%), and *Bacillus amyloliquefaciens* subsp*. palnta* FZB42T (97.49%). The neighbour‐joining phylogenetic tree further confirmed that the strain N11‐8 was phylogenetically related to the genus *Bacillus* (Fig. 1).

**Figure 2:**
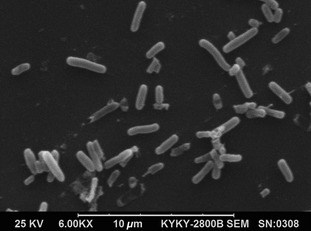
Scanning electron micrographs of Antarctic marine *Bacillus* N11‐8. Scale Bar = 10 *μ*m.

In addition to the features that define the strain, the following characteristics are observed. The morphological characters of the cells were clearly visualized by SEM. Cells are ovoid or irregular short rods, approximately 0.5–2.5 mm long and 0.5–0.8 mm wide (Fig. 2). The colonies are uniformly round, 0.8–2.1 mm in diameter, and have smooth, glossy, and mucoid appearance, with texture like cream, white, and translucence. Oxidase is negative.

The temperature range for growth was 20–30°C. The pH range for growth was 7.0–8.0. According to API 20E and API 20NE: Urease and Voges–Proskauer reactions were positive; They were negative for *β*‐galactosidase, arginine dihydrolase, lysine decarboxylase, ornithine decarboxylase, tryptophan deaminase, and gelatinase; citrate was not utilized; neither H_2_S or indole was produced; acid was produced from d‐glucose, melibiose, l‐arabinose, and sucrose, but not from d‐mannitol, inositol, d‐sorbitol, l‐rhamnose, and amygdalin. The fermentation media contained d‐glucose, sucrose, melibiose and l‐arabinose; it is negative for assimilation experiments: l‐arabinose, *N*‐acetylglucosamine, potassium gluconate, capric acid, adipic acid, malic acid, trisodium citrate, d‐mannitol, d‐mannose, maltose, and phenylacetic acid. According to API ZYM, the isolated strain was positive for alkaline phosphatase, lipase (C14), leucine arylamidase, valine arylamidase, cystine arylamidase, trypsin, chymotrypsin, acid phosphatase, naphthol‐AS‐BI‐phosphohydrolase, *α*‐galactosidase, *β*‐glucuronidase, *α*‐glucosidase, *β*‐fucosidase, and *N*‐acetylglucosaminidase; and negative for esterase (C4), esterase lipase (C8), *β*‐glucosidase and *α*‐mannosidase.

### Fermentation and preparation of the fermentation products of Antarctic marine *Bacillus* sp. N11‐8

About 30 L of production media was fermented. The products of different molecular weight <1 kDa, 1–30 kDa, 30–50 kDa and >50 kDa were separated using ultrafiltration. The fermentation products of molecular weight of 1–30 kDa showed highest antitumor activity on BEL‐7402 cells as determined by MTT assay and was chosen for further studies.

### The cytotoxicity of the fermentation products of Antarctic marine *Bacillus* sp. N11‐8 against tumor cells

**Figure 3:**
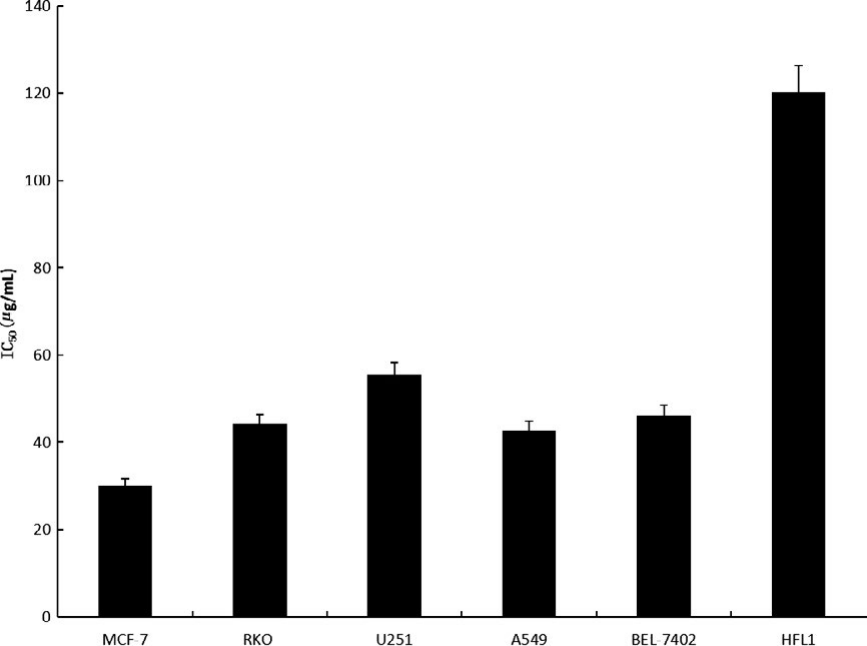
Cytotoxicity of the fermentation products of Antarctic marine *Bacillus* sp. N11‐8 on tumor cells. BEL‐7402 Human hepatocellular carcinoma cells, RKO human colon carcinoma cells, A549 human lung carcinoma cells, U251 human glioma cells, MCF‐7 human breast carcinoma cells, and HFL1 human normal fibroblast lung cells were treated with certain concentrations of the fermentation products of N11‐8 for 48 h. The cell inhibitory rate was determined usingthe MTT assay as described in Materials and methods. Data were presented as means ± SD of three independent experiments; *P < 0.05, compared with HFL1 human normal fibroblast lung cells.

**Figure 4:**
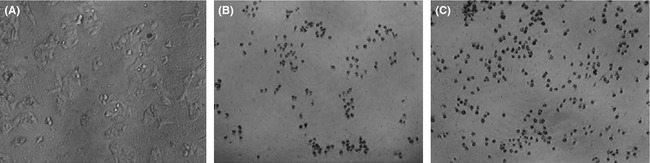
Morphological changes in BEL‐7402 cells induced by fermented active products of the Antarctic marine *Bacillus* sp. N11‐8 (A) Negative control; (B) Fermented active products of the Antarctic marine *Bacillus* sp. N11‐8; (C) Positive control.

Marine microorganisms are a major source of natural products (Waters et al. 2010; Xiong et al. 2013). Anticancer *ε*‐poly‐l‐lysine (*ε*‐PL) was produced by a marine *Bacillus subtilis* sp. isolated from sea water in Alexandria (El‐Sersy et al. 2012). Sungsanpin, a new 15‐amino‐acid peptide, was discovered from a Streptomyces species isolated from deep‐sea sediment collected off Jeju Island, Korea. Sungsanpin displayed inhibitory activity in a cell invasion assay with the human lung cancer cell line A549 (Um et al. 2013). The cytotoxicity of the fermentation products of N11‐8 (molecular weight 1–30 kDa) was determined by MTT assay. As shown in Figure 4, the fermentation products of N11‐8 reduced the viability of five selected cancer cell lines in a dose‐dependent manner. The IC_50_ values of BEL 7402, RKO, A549, U251 and MCF‐7 cells were detected. For instances, the IC_50_ value is 30.15 ± 1.51 *μ*g/mL on MCF‐7 cells. However, relatively low cytotoxicity was detected against HFL1 cells (Fig. 3). The MTT results suggested that the fermentation products of Antarctic marine *Bacillus* sp. N11‐8 are expected to be used for the treatment of liver cancer, glioma, lung cancer, and breast cancer. Additionally, the inhibition effect of the fermentation products of N11‐8 on cell viability exhibited some specificity to tumor cells.

### Phase contrast microscopy analysis of the effect of the fermentation products of Antarctic marine *Bacillus* sp. N11‐8 on the morphology of hepatoma cells

MTT results showed that the fermentation products of Antarctic marine *Bacillus* sp. N11‐8 have an inhibitory activity on BEL‐7402 cells in vitro. Therefore, the morphological changes of tumor cells caused by the fermentation products were observed under phase contrast microscope. In comparison with control groups (Fig. 4A), after the treatment with the fermentation products, cells became round, and then shrank, and finally ruptured (Fig. 4B); in the positive control group (50 *μ*g/mL mitomycin C), cells displayed similar changes (Fig. 4C). These results showed that the fermentation products of Antarctic marine *Bacillus* sp. N11‐8 can change the morphology of BEL‐7402 cells, similar to mitomycin C.

### The pH stability of the fermentation products of Antarctic marine *Bacillus* sp. N11‐8

**Figure 5:**
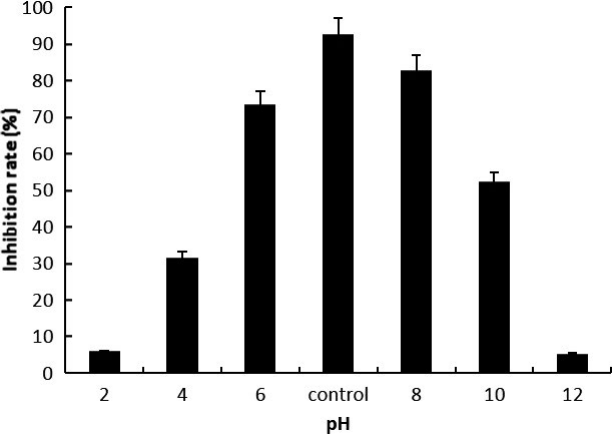
PH stability of the fermented active products of Antarctic marine *Bacillus* sp. N11‐8. Data were presented as means ± SD of three independent experiments; *P < 0.05, compared with the controls.

The pH stability of the fermentation products of Antarctic marine *Bacillus* sp. N11‐8 is shown in Figure 5. The antitumor activity of the fermentation products of Antarctic marine *Bacillus* sp. N11‐8 changed little between pH 6 and pH 9 (no significant difference). When pH is lower than 6 or higher than 9, the cytotoxicity was Gradually decreased. The active component was stable in weak acidic, neutral and alkaline conditions, but was sensitive to strong acidic and alkaline conditions. Therefore, purification should be kept in a neutral, weak acidic, or weak basic condition.

### The thermal stability of the fermentation products of Antarctic marine *Bacillus* sp. N11‐8

**Figure 6:**
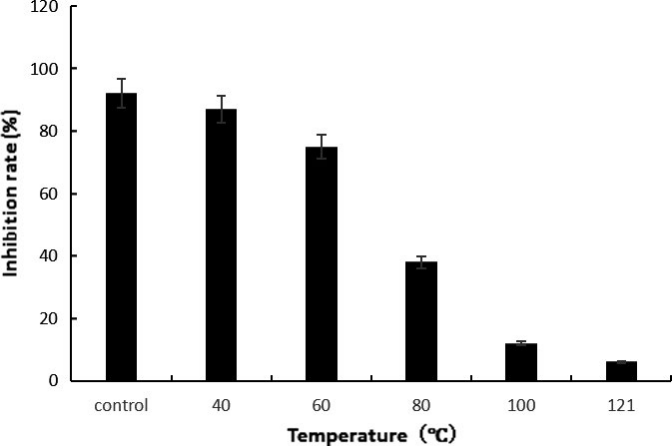
Thermal stability of the fermented active products of the Antarctic marine *Bacillus* sp. N11‐8. Data were presented as means ± SD of three independent experiments; *P < 0.05, compared with the controls.

The fermentation products of Antarctic marine *Bacillus* sp. N11‐8 has good thermal stability, as shown in Figure 6. Compared with the controls, 60°C had no effect on the antitumor activity of the fermentation products. But along with the increase in temperature, its antitumor activity decreased gradually, which may be due to the change in the structure of active substances in the fermentation products.

## Conclusions

Most Antarctic marine strains obtained in this study may be developed as promising sources for discovery of antitumor bioactive substances. Fermented active products from the *Bacillus* sp. N11‐8 is expected to be used in tumor prevention and treatment, which has important research and application value.

## Conflict of Interest

The authors declare no conflict of interest.
